# DNA integrity and viability of testicular cells from diverse wild species after slow freezing or vitrification

**DOI:** 10.3389/fvets.2022.1114695

**Published:** 2023-01-16

**Authors:** Patricia Peris-Frau, Julia Benito-Blanco, Eva Martínez-Nevado, Adolfo Toledano-Díaz, Cristina Castaño, Rosario Velázquez, Belén Pequeño, Belén Martinez-Madrid, Milagros C. Esteso, Julián Santiago-Moreno

**Affiliations:** ^1^Departamento de Reproducción Animal, Instituto Nacional de Investigación y Tecnología Agraria y Alimentaria-CSIC, Madrid, Spain; ^2^Zoo-Aquarium Madrid, Madrid, Spain; ^3^Department of Animal Medicine and Surgery, Faculty of Veterinary Medicine, Complutense University of Madrid, Madrid, Spain

**Keywords:** testicular tissue cryopreservation, freezing-thawing, germ cell survival, wildlife, endangered species testes, germ cell DNA integrity, germplasm preservation

## Abstract

**Introduction and objective:**

Cryopreservation of testicular tissues offers new possibilities to protect endangered species, genetically valuable individuals or even the fertility potential of prepubertal individuals who have died unexpectedly. However, the use of this technique still remains a challenge. In this study, slow freezing and vitrification of testicular tissue was investigated to find out which cryopreservation method could better preserve the viability and DNA integrity of testicular germ cells in diverse wild species.

**Methods:**

Testes were obtained post-mortem from 18 artiodactyls (wild boar, roe deer, dwarf goat, mhor gazelle, European mouflon, African forest buffalo, Malayan tapir, dorcas gazelle, Iberian ibex, gnu, red river hog), 5 primates (colobus monkey, capuchin monkey, mandrill), 8 carnivores (gray wolf, Persian leopard, binturong, European mink, American black bear, suricata), and 2 rodents (Patagonian mara). The testicles belonged to adult individuals and were cut into small pieces and cryopreserved by needle immersed vitrification or uncontrolled slow freezing using a passive cooling device. After warming or thawing, testicular tissues were enzymatically digested and two germ cell types were differentiated based on their morphology: rounded cells (spermatogonia, spermatocytes, and early spermatids) and elongated cells (elongated spermatids and spermatozoa). Cell viability was assessed by SYBR-14/propidium iodide while DNA fragmentation by TUNEL assay with fluorescence microscope.

**Results and discussion:**

Our preliminary results revealed that our uncontrolled slow freezing method better preserved the viability and DNA integrity of elongated cells than vitrification. Such trend was observed in all species, being significant in artiodactyls, carnivores, and primates. Similarly, the viability and DNA integrity of rounded cells was also better maintained in primates by uncontrolled slow freezing, while in carnivores, vitrification by needle immersion showed better results in this type of cells. In artiodactyls and rodents both techniques preserved the viability of rounded cells in a similar manner, although the DNA integrity of these cells was greater after needle immersed vitrification in artiodactyls.

**Conclusions:**

In conclusion, the effectiveness of each cryopreservation method is affected by the phylogenetic diversity between species and cell type.

## 1. Introduction

Each year, more species are in risk of extinction, especially wild species, due to habitat loss, inbreeding problems, climate change or overexploitation (1). Cryopreservation of testicular tissue has recently emerged as a promising technique for preserving biodiversity and also genetically valuable males (2). This technique is particularly interesting when sperm collection is not possible, such as in prepubertal animals that die unexpectedly, strongly seasonal species who die in non-breeding season, males suffering from some pathology related to azoospermia and even in prepubertal patients who will receive gonadotoxic therapies after cancer detection (3–6). Despite the latest advances, the use of this technique still remains a challenge due to the complex structure of testes, the heterogeneous response of multiple germ cells to cryopreservation and the differences between species (4, 7, 8).

Testicular tissues contain somatic cells, such as Sertoli, Leydig, and myoid cells and also many types of germ cells, ranging from spermatogonia, spermatocytes, and spermatids to spermatozoa (4). Following cryopreservation, testicular fragments can be grafted into a suitable host or early germ cells, like spermatogonia, can be extracted from testicular tissues and *in vitro* culture to resume, in both cases, spermatogenesis and produce mature spermatozoa for assisted reproductive technologies (3, 5, 6).

Slow freezing and vitrification are the main techniques used for cryopreservation of testicular tissues. However, there are different protocols developed for both techniques. For instance, slow freezing can be achieved by controlling all the time the cooling rate (controlled slow freezing) or without controlling (uncontrolled slow freezing). The uncontrolled slow freezing requires a small passive cooling device to achieve a cooling rate of 1°C/min, which is less expensive than a programmable freezer (controlled slow freezing), easier and can be used outside the lab (4). For testicular tissue vitrification, the methods or protocols most employed are conventional vitrification, solid surface vitrification, and needle immersed vitrification (9–11). One of the advantages of slow freezing is the lower concentration of cryoprotectants in comparison to vitrification either in controlled or uncontrolled method, which considerably decreases the cytotoxic effects of these agents (12). However, the procedure of vitrification, in general, is faster than slow freezing and considerably reduces intracellular and extracellular ice formation by using high concentration of cryoprotectants and ultra-rapid cooling rates (>106°C/min) (8). In addition, this latter technique is cost-effective and an excellent option for wildlife species since it can be used under field conditions.

Notwithstanding, the best technique for testicular cryopreservation in different species is still unclear. Previous studies have shown variable results when different cryopreservation techniques were compared. In fact, the differences are also evident when different protocols or methods are used within the same technique and compared (10, 11). In boars and cats, slow freezing and vitrification showed similar results in terms of testicular cell survival (2, 13, 14). Conversely, in domestic rodents and dogs, vitrification was superior to slow freezing in preserving testicular cell integrity (2, 15–17). In other studies, fast freezing led to greater testicular sperm viability than slow freezing or vitrification in cats (18, 19), while slow freezing using dimethylsulfoxyde, ethylene glycol, and trehalose provided better results than vitrification in dogs (2). In ram, slow freezing better preserved testicular morphology than vitrification (20). Among wild species, comparative studies have been conducted in wild boar (17), red-rumped agouti (11), collared peccary (10), gray wolf (9), jungle cat, lion, leopard, rusa deer, fea's muntjac, and sumatran serow (21) but there are many wildlife species in which comparison of cryopreservation methods are still required to better understand the species-specific differences that lead to structural and functional germ cell damage. In wild boar and collared peccary both techniques are equally effective (10, 17). Furthermore, two vitrification methods were also compared in the latter species, obtaining better results with conventional vitrification than with solid surface vitrification (10). In red-rumple agouti, vitrification was superior than slow freezing (11). Contrary to collared peccary, solid surface vitrification in red-rumple agouti testes provided greater results than conventional vitrification (11). In gray wolf, slow freezing demonstrated a superior ability to protect testes than vitrification (9). In the other three felids and three ungulates, fast freezing better preserved the integrity of testicular sperm than slow freezing but testicular morphology and intra-tubular cells were damaged (21).

Obtaining samples from wild species is difficult, essentially from endangered species, because of the limited accessibility or availability of animals. Therefore, it would be ideal to adapt testicular cryopreservation protocols for use in a large number of species that belongs to the same taxonomic group with minor modifications for each species (20). Such idea requires to test protocols in diverse closely related species to demonstrate their cross-species adaptability. With this background, our study aimed to determine in a large variety of wild species which of the two methods for preserving testicular tissues (uncontrolled slow freezing or needle immersed vitrification) offers a better protective effect on germ cells viability and DNA integrity in different taxonomic groups.

## 2. Materials and methods

All reagents were purchased from Merck (Darmstadt, Germany) unless otherwise specified.

### 2.1. Animals and testes collection

**Figure 1:**
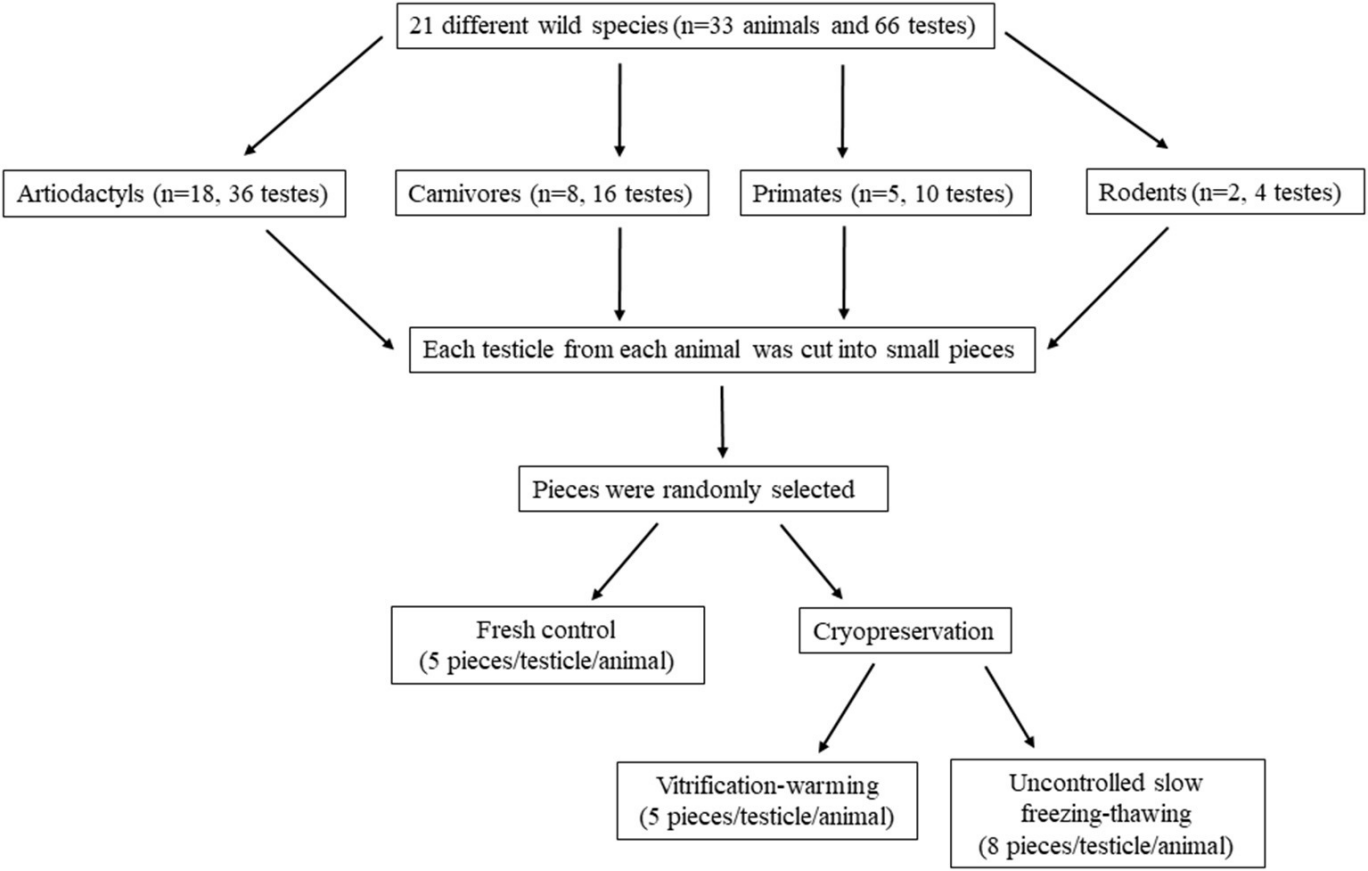
Flowchart showing the experimental design.

Testes (*n* = 66) were obtained postmortem from adult wild species grouped according to their order in artiodactyls (*n* = 18), carnivores (*n* = 8), primates (*n* = 5), and rodents (*n* = 2). All animals (*n* = 33) were provided by the Madrid Zoo-Aquarium and Madrid Regional Government's Wild Animal Recovery Centre. Individual information, including the age at death, cause of death, reproductive disorders, and the time elapsed between death and laboratory is shown in Table 1. The testes were kept within the scrotum in plastic bags at 4°C until their reception in the laboratory. Once there, the scrotal sac, tunica albuginea and the attached epididymis were removed. The testicular parenchyma was then cut into small pieces (2 x 2 x 2 mm, in all species and in all cryopreservation methods) in an initial holding solution (medium TCM 199) and washed three times for 30 s in the basal medium used for cryopreservation, Dulbecco's Modified Eagle Medium with Nutrient Mixture F-12 (DMEM F-12) with 20% Fetal Bovine Serum (FBS). Eight randomly fragments from each testicle and animal were processed as fresh samples while eight fragments were frozen in a slow passive cooling device (uncontrolled slow freezing) and other five fragments were vitrified. The experimental design is summarized in Figure 1.

**Table 1:** General information about the animals (*n* = 33) used in the study.

**Species**	**Scientific name**	**Order**	**Number of animals**	**Age (years)**	**Cause of death**	**Time from death to laboratory (h)**	**Reproductive disorders**
Wild boar	*Sus scrofa*	Artiodactyla	3	5–8	Unknown	12–24	–
Roe deer	*Capreolus capreolus*	Artiodactyla	1	4	Bone fracture	20	–
Dwarf goat	*Capra aegagrus hircus*	Artiodactyla	2	6–7	Unknown	18	–
Mhorr gazelle	*Nanger dama mhorr*	Artiodactyla	1	3	Anesthetic complication	28	–
European mouflon	*Ovis musimon*	Artiodactyla	2	8–9	Surgery complication and euthanasia	24–48	–
African forest buffalo	*Syncerus caffer nanus*	Artiodactyla	1	7	Bone fracture	15	–
Malayan tapir	*Acrocodia indica*	Artiodactyla	1	16	Unknown	31	–
Dorcas gazelle	*Gazella dorcas osiris*	Artiodactyla	2	1–3	Bone fractures	14	–
Iberian ibex	*Capra pyrenaica*	Artiodactyla	2	7–9	Vehicle collision	24–12	–
Gnu	*Connochaetes gnou*	Artiodactyla	1	4	Septicemia	34	–
Red river hog	*Potamochoerus porcus pictus*	Artiodactyla	1	14	Aging	18	Azoospermic
Colobus monkey	*Piliocolobus kirkii*	Primate	2	15–16	Unknown	15–30	–
Capuchin monkey	*Cebus apella*	Primate	2	1–3	Unknown	18–15	One of them was oligospermic
Mandrill	*Mandrillus sphinx*	Primate	1	16	Metastasis	48	–
Gray wolf	*Canis lupus occidentalis*	Carnivora	1	8	Head trauma and euthanasia	10	Azoospermic
Persian leopard	*Panthera pardus saxicolor*	Carnivora	1	12	Aging	16	–
Binturong	*Arctictis binturong*	Carnivora	1	18	Aging	38	–
European mink	*Mustela lutreola*	Carnivora	1	2	Vehicle collision	36	–
American black bear	*Ursus americanus*	Carnivora	1	14	Euthanasia	24	Azoospermic
Suricata	*Suricata suricatta*	Carnivora	3	10–13	Septicemia and aging	10–20	–
Patagonian mara	*Dolichotis patagonum*	Rodentia	2	12–13	Aging	10–15	–

### 2.2. Slow freezing-thawing

Slow freezing was performed following the protocol described by Picazo et al. (17). Eight tissue fragments from each testicle were transferred to a 2 ml cryovial with 0.5 ml of DMEM F-12 supplemented with 20% FBS and 2.8 M dimethylsulfoxyde (DMSO). The cryovials were then placed into a CoolCell^®^ container (Corning, Arizona, USA) at −80°C overnight to achieve a cooling rate of −1°C/min and subsequently stored in a liquid nitrogen container (−196°C). Cryovials were thawed after maintaining them for 1 min at 37°C. Thawed pieces were progressively transferred to 3 Petri dishes containing DMEM F-12, 20% FBS and decreasing concentrations of DMSO (1, 0.5, and 0 M) for 5 min each at RT.

### 2.3. Vitrification-warming

Five tissue fragments from each testicle were threaded onto an acupuncture needle (0.18 x 30 mm; medical Device Co., Ltd. Wujiang, China) and plunged into DMEM F-12 with 1.06 M DMSO and 1.35 M ethylene glycol according to Picazo et al. (17). After 10 min of equilibration, the needles were immersed in DMEM F-12 with 2.1 M DMSO, 2.7 M ethylene glycol, and 0.5 M sucrose for 2 min. Thereafter, the needles were plunged into liquid nitrogen and immediately placed into pre-cooled 2 ml straws (Cryo BioSystem, Saint Ouen Sur Iton, France). The straws were sealed and stored in a nitrogen container. For warming, the needles were immersed in DMEM F12 with 20% FBS and 1 M sucrose during 5 s at 50°C after removing them from straws. Tissue fragments were then progressively transferred to 3 Petri dishes containing DMEM F-12, 20% FBS and decreasing concentrations of sucrose (0.5, 0.25, and 0 M) for 5 min each at RT.

### 2.4. Testicular tissue disaggregation

Testicular cells were extracted from fresh, frozen/thawed or vitrified/warmed tissue fragments by combining mechanical mincing with enzymatic digestion following the protocol described by Li et al. (22). The pieces were incubated in 2 ml of DMEM F-12 with 14 mg collagenase IV (Gibco, Life Technologies Corporation, NY, USA) for 40 min at 37°C and every 5 min mixed with trimmed pipette tips. After washing (1,000 g x 5 min) with DMEM F-12 and removing the supernatant, the pellet was incubated in 1 ml of 0.05% trypsin-EDTA at 37°C for 5 min (17). The reaction was stopped with 1 ml of DMEM F-12 supplemented with 10% FBS, and then centrifuged (1,000 g x 5 min). The pellet was resuspended in 50 μl of DMEM F-12 with 10% FBS. Two well-differentiated cell types were identified based on their morphology: rounded cells (RC; which include spermatogonia, spermatocytes, and early spermatids) and elongated spermatids together with spermatozoa (ESS). Non-rounded cells with irregularly shaped nuclei compatible with Sertoli cells and polygonal cells with large round nuclei compatible with Leydig cells were not analyzed. The morphological classification of cells was subjectively made by two experienced evaluators based on the shape of cells (cytoplasm and nucleus) after observing them in the microscope using brightfield view and fluorescence filters. Although most rounded cell corresponded to germ cells, other cell types were also present but in a lower number, such as Leydig cells, which can be confused with rounded germ cells since they possess a round nuclei and were also stained.

### 2.5. Cell viability

**Figure 2:**
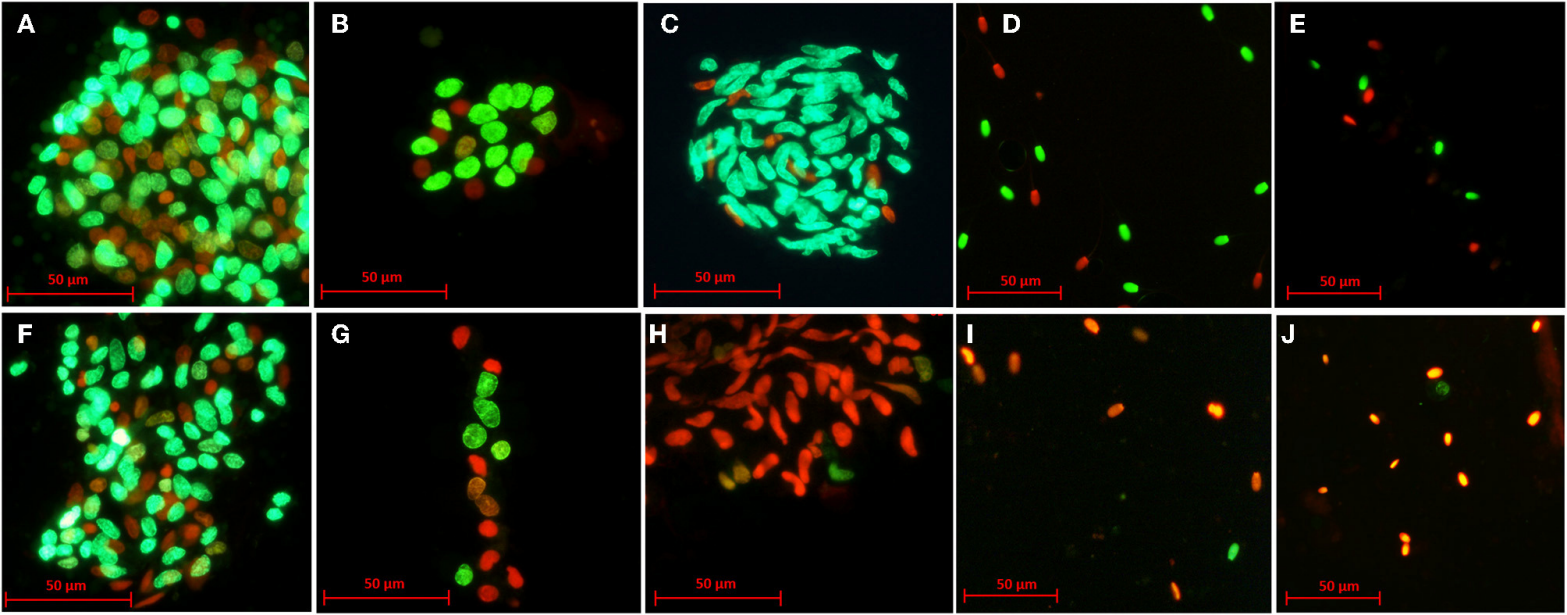
Representative images of the viability of testicular cells after slow freezing-thawing **(A–E)** and vitrification-warming **(F–J)**. Green cells (SYBR-14+) represent live cells, while orange or red cells (PI+) represent dead cells. Rounded germ cells from an artiodactyl (dorcas gazelle) after slow freezing-thawing and vitrification-warming **(A, F)**, rounded germ cells from a primate (Mandrill) after slow freezing-thawing and vitrification-warming **(B, G)**, elongated spermatids from a carnivore (Persian leopard) after slow freezing-thawing and vitrification-warming **(C, H)**, spermatozoa from an artiodactyl (European mouflon) after slow freezing-thawing and vitrification-warming **(D, I)**, spermatozoa from a carnivore (European mink) after slow freezing-thawing and vitrification-warming **(E, J)**.

Cell viability was determined with the Live/Dead™ Sperm Kit (Life Technologies Corporation, Oregon, USA) as has been previously described by Picazo et al. (17). Briefly, 10 μl of cell suspension was incubated with 2 μl of SYBR-14 (0.05 mM) and 1 μl of propidium iodide (2.4 mM) for 5 min/each at 5°C in the dark. At least 200 cells/sample were classified as live (green fluorescence, SYBR-14 +; wavelength: 450–490 nm) or dead (red fluorescence, PI +; wavelength: 450–490 nm) using a fluorescence microscope (Eclipse E200, Nikon, Japan) at 400x (Figure 2).

### 2.6. DNA integrity

**Figure 3:**
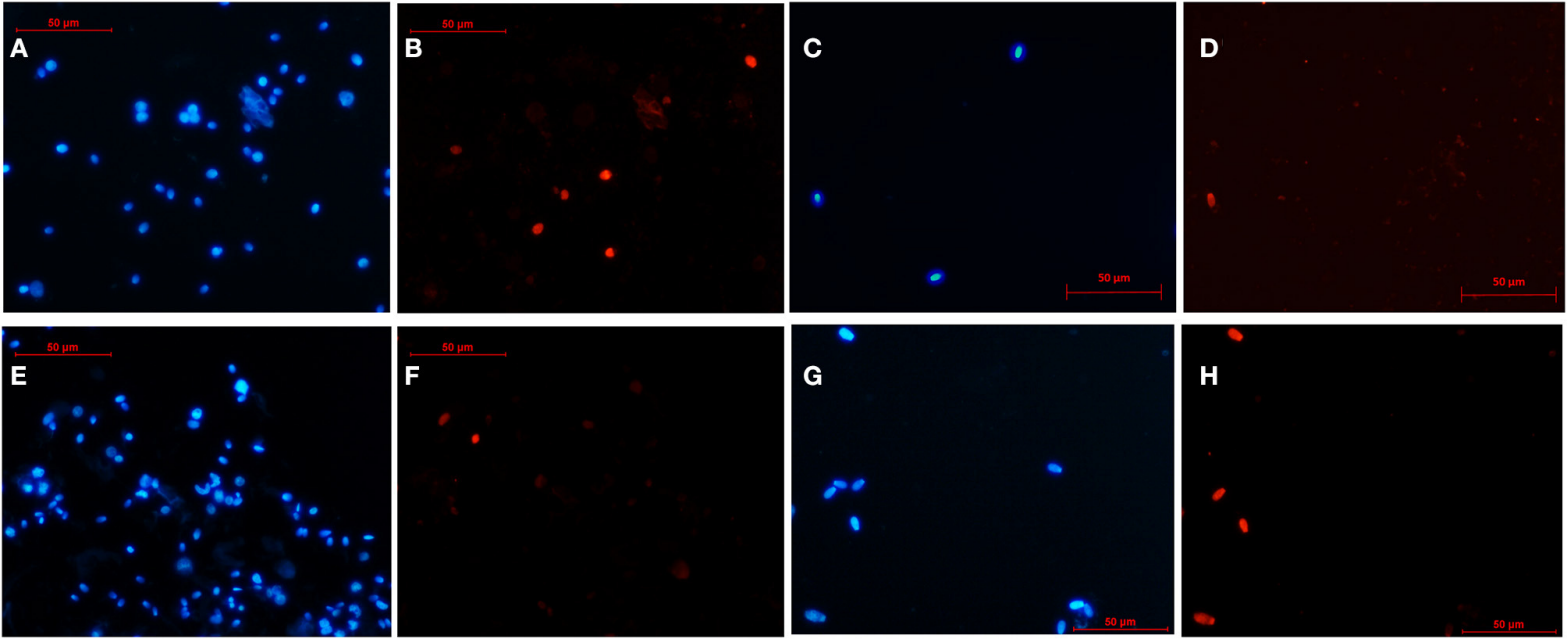
Representative images of the DNA integrity of testicular cells after slow freezing-thawing **(A–D)** and after vitrification-warming **(E–H)**. The nucleus of testicular cells was stained with Hoechst (blue fluorescence) and those showing DNA fragmentation also emitted red fluorescence (TUNEL+). Rounded and elongated germ cells from a primate (Capuchin monkey) after slow freezing-thawing **(A, B)**, spermatozoa from a rodent (Patagonian mara) after slow freezing-thawing **(C, D)**, rounded and elongated germ cells from a carnivore (Suricata) after vitrification-warming **(E, F)**, spermatozoa from an artiodactyl (Iberian ibex) after vitrification-warming **(G, H)**.

DNA integrity was evaluated with the terminal deoxynucleotidyl transferase dUTP nick end labeling assay (TUNEL) using the *In Situ* Cell Death Detection Kit (Roche, Basel, Switzerland) according to the manufacturer's instructions with some modifications introduced by Cardoso et al. (23). Briefly, 40 μl of cell suspension was fixed in 40 μl of 4% paraformaldehyde in PBS. The fixed sample (10 μl) was spread onto a glass slide and allowed to dry. After permeabilizing with 0.1% of Triton X-100 in PBS for 5 min and washing with PBS, DNA fragmentation was detected by incubating the slide with 10 μl of terminal deoxynucleotidyl transferase (TdT)–fluorescent-labeled nucleotide mix for 1 h in a dark humidified chamber at 37°C. The slides were rinsed with PBS and then counterstained with 0.1 mg/ml of Hoechst 33342 for 5 min to visualize total DNA. A drop of aqueous mounting medium (Fluoromont^®^) was placed on the slides. At least 200 cells/sample were counted using a fluorescence microscope at 400 × (Eclipse E200; Figure 3). Cells with fragmented DNA showed red fluorescence (TUNEL +, wavelength: 510–560 nm) and also blue fluorescence (Hoechst +), while cells with intact DNA (TUNEL –) only showed blue fluorescence (Hoechst +; wavelength: 361–497 nm).

### 2.7. Statistical analysis

All statistical analyses were performed using Statistica software v.13.0 (StatSoft Inc., Tulsa, OK, USA). Data were tested for normality with the Shapiro-Wilk test and arcsine-transformed to meet the requirement. The effect of treatment (fresh, slow freezing, vitrification) and cell type (rounded cells, elongated cells) on cell viability and DNA integrity was evaluated using ANOVA and the Tukey– Kramer test as a *post-hoc* test. Significance was adjusted to *P* < 0.05. The interaction between species and treatment was included in the model and was significant in all cases. For this reason, a second ANOVA was performed to analyze within each order of animals (artiodactyla, rodentia, carnivore, and primates) the influence of cryopreservation method on the viability and DNA integrity of rounded cells and elongated spermatids/spermatozoa. The species were grouped according to their order because in some cases there is one individual of each species while in others there are more. Values are expressed as means ± SEM.

## 3. Results

### 3.1. General effect of slow freezing and vitrification on testicular germ cells in all wild species

**Figure 4:**
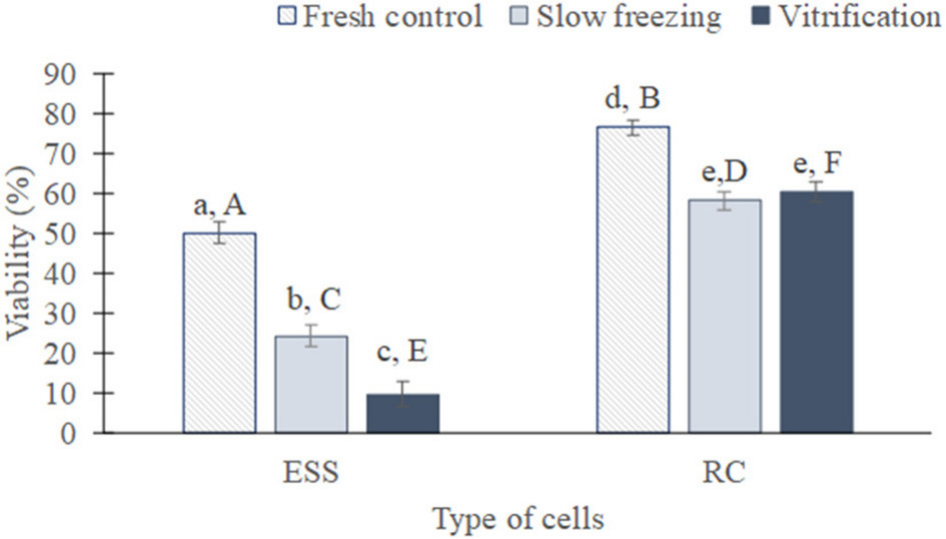
Global viability of testicular cells (mean from all species ± SEM) from fresh, slow frozen-thawed and vitrified-warmed samples. Different lowercase letters represent differences (*P* < 0.05) between treatments for each cell type. Different capital letters represent differences (*P* < 0.05) between cell types in each treatment. ESS, elongated spermatids and spermatozoa; RC, rounded cells (spermatogonia, spermatocytes, early spermatids).

**Figure 5:**
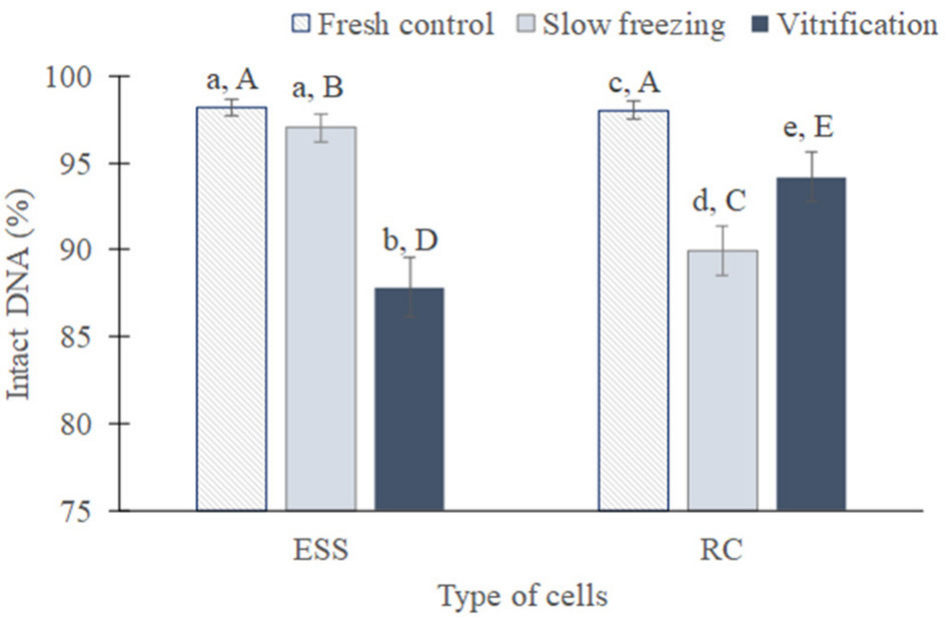
Global DNA integrity of testicular cells (mean from all species ± SEM) from fresh, slow frozen-thawed and vitrified-warmed samples. Different lowercase letters represent differences (*P* < 0.05) between treatments for each cell type. Different capital letters represent differences (*P* < 0.05) between cell types in each treatment. ESS, elongated spermatids and spermatozoa; RC, rounded cells (spermatogonia, spermatocytes, early spermatids).

In a first global analysis, all the species were studied together and the cryopreservation techniques were compared between them and with fresh samples to determine their impact on the viability and DNA integrity of germ cells (Figures 4, 5).

As expected, the proportion of viable rounded cells and elongated spermatids/spermatozoa was superior before than after cryopreservation, regardless of the technique used (*P* < 0.01 in both cases, Figure 4). After cryopreservation, the percentage of viable elongated spermatids/spermatozoa was greater in slow frozen/thawed samples (24.1 ± 2.6%) than vitrified/warmed (9.6 ± 2.9%; *P* < 0.05). In contrast, the proportion of viable rounded cells was similar between both methods (58.5 ± 2.3 vs. 60 ± 2.4%; *P* = 0.49). When both type of cells were compared, rounded cells exhibited a higher viability than elongated spermatids/spermatozoa either in fresh samples (76.3 ± 1.8 vs. 50 ± 2.7%) or slow freezing and vitrification (*P* < 0.01 in all cases).

Regarding DNA fragmentation (Figure 5), the percentage of elongated spermatids/spermatozoa with intact DNA was similar between fresh (98.1 ± 0.4%) and slow frozen/thawed samples (97 ± 0.8%; *P* = 0.47), but was considerably reduced after vitrification (87.8 ± 1.7%) compared to fresh and slow frozen/thawed samples (*P* < 0.05). In rounded cells, DNA integrity decreased after cryopreservation (*P* < 0.05) compared to fresh samples (98 ± 0.9%), being lowest in slow frozen/thawed samples (89.9 ± 1.4 vs. 94.2 ± 1.4% vitrified/warmed; *P* < 0.05). In relation to cell type, DNA integrity was lower in elongated spermatids/spermatozoa in comparison to rounded cells after vitrification, occurring the opposite in slow freezing (*P* < 0.01 in both cases). In fresh conditions, DNA integrity was similar between both type of cells (*P* = 0.88).

### 3.2. Impact of slow freezing and vitrification on testicular germ cells within each taxonomic group

**Figure 6:**
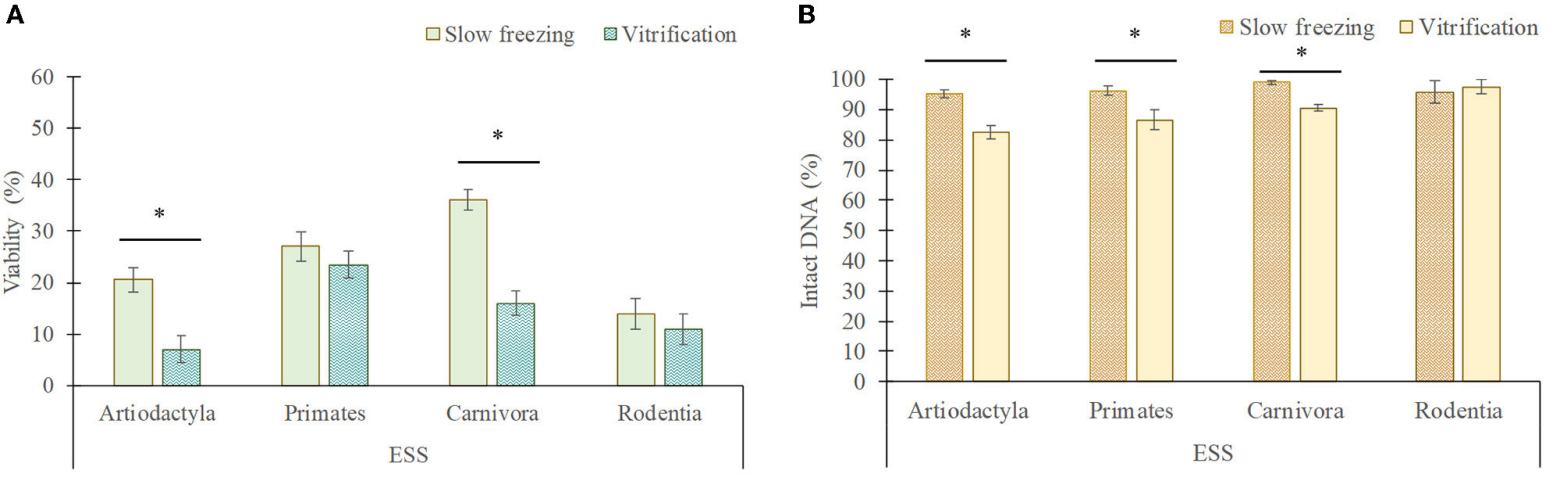
Viability **(A)** and DNA integrity **(B)** of elongated spermatids and spermatozoa (ESS) in each taxonomic group (mean ± SEM) after slow freezing-thawing and vitrification-warming of testicular tissues. *Indicate differences (*P* < 0.05) between cryopreservation methods.

**Figure 7:**
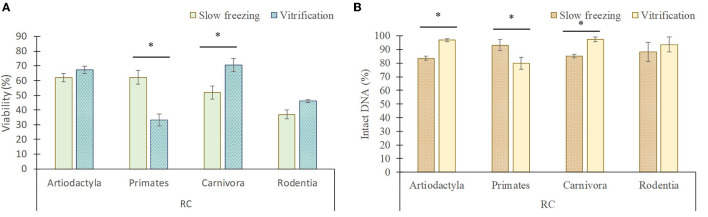
Viability **(A)** and DNA integrity **(B)** of rounded cells (RC; spermatogonia, spermatocytes, early spermatids) in each taxonomic group (mean ± SEM) after slow freezing-thawing and vitrification-warming of testicular tissues. *Indicate differences (*P* < 0.05) between cryopreservation methods.

There is a wide diversity between the species evaluated, which implies physiological and also morphological differences, specially related to the testicular architecture (24). Therefore, the species were grouped according to their order in a second analysis to determine whether there is a different sensitivity to the cryopreservation techniques between the taxonomic groups (Figures 6, 7).

In artiodactyls, the viability (20.5 ± 2.4% frozen/thawed vs. 7 ± 2.5% vitrified/warmed) and DNA integrity (95.3 ± 1.2% frozen/thawed vs. 81.5 ± 2.3% vitrified/warmed) of elongated spermatids/spermatozoa was better maintained by slow freezing than by vitrification (*P* < 0.05). However, vitrification showed a greater preservation of the DNA integrity of rounded cells than slow freezing (96.8 ± 1.1 vs. 83.5 ± 1.7%; *P* < 0.05), although no differences in the proportion of viable rounded cells were detected between both techniques (*P* > 0.05).

In primates, slow freezing and vitrification maintained the viability of elongated spermatids/spermatozoa in a similar manner (*P* > 0.05), although their DNA integrity was lower after vitrification than after slow freezing (86.5 ± 3.2 vs. 96.2 ± 1.6%). In addition, vitrification considerably reduced the viability (23.4 ± 2.5 vs. 62.2 ± 4.6%) and DNA integrity (80 ± 4.4 vs. 93.2 ± 4%) of rounded cells compared to slow freezing (*P* < 0.05).

In carnivores, the viability (36 ± 1.9 vs. 16 ± 2.4%) and DNA integrity (99 ± 0.7 vs. 88.5 ± 1%) of elongated spermatids/spermatozoa was better preserved by slow freezing, while vitrification better maintained the viability (70.6 ± 4.3 vs. 51.8 ± 4.6%) and DNA integrity (97.3 ± 1.6 vs. 85.1 ± 1.3%) of rounded cells (*P* < 0.05).

In rodents, the viability and DNA integrity of elongated spermatids/spermatozoa and rounded cells was similar between both techniques (*P* > 0.05), although the proportion of live rounded cells tended to decrease after slow freezing in comparison to vitrification (37 ± 3.2 vs. 45 ± 1.9%; *P* = 0.07).

## 4. Discussion

Our preliminary results showed that the effectiveness of each testicular cryopreservation method is affected by the phylogenetic diversity between species and the germ cell type (rounded vs. elongated cells). Although most testicular rounded cells corresponded to germ cells (spermatogonia, spermatocytes, and early spermatids), other cell types were present in a lower number and stained, such as Leydig and Sertoli cells, but the exclusion of Sertoli cells was easier due to their bigger size, cytoplasm, and the irregularly shaped nuclei. In line with a previous study (17), rounded cells showed, in general, a greater cryoresistance than elongated spermatid and spermatozoa to both testicular cryopreservation techniques. Our findings are also supported by Lee et al. (25) who found that spermatogonia were more resistant to cryodamage than other germ cells. One possible explanation could be the different sensitivity of diverse germ cells to cryoprotectants as well as their differences in nucleus compaction and cytoplasm ratio (14, 17). Glycerol has been found to be more effective for spermatozoa and spermatids, while dimethylsulfoxyde and ethylene glycol for spermatogonia and spermatocytes (19, 26). In our study, the cryoprotectant used for slow freezing was dimethylsulfoxyde and for vitrification dimethylsulfoxyde, ethylene glycol and sucrose which would explain the greater viability of rounded cells compared to elongated spermatids/spermatozoa. On the other hand, the antioxidant defense of spermatids and spermatozoa is quite scarce due to their reduced cytoplasm (27), being more vulnerable to suffer oxidative and osmotic stress during freezing/thawing or vitrification/warming than rounded cells. However, the nucleus of elongated spermatids and spermatozoa is more compacted than in early germ cells because of the replacement of histones by protamines during spermatogenesis (28). Such nuclear organization provides a better protection of the DNA against cryodamage, explaining our greater results during slow freezing for elongated cells compared to rounded ones. After vitrification, the opposite effect was observed on DNA integrity. One possible reason could be the high concentration of cryoprotectants used in this techniques, since it has been reported to cause serious osmotic and chemical modifications in spermatozoa (29), reducing their quality possibly by the damage inflicted on plasma membrane and DNA.

Regarding the effect of the testicular cryopreservation method on each germ cell type, slow freezing was less harmful for elongated spermatid and spermatozoa than vitrification, which is consistent with previous reports in ejaculated spermatozoa (30–32). In fact, this trend was maintained through the different taxonomic groups, being significant in artiodactyls and carnivores for viability and DNA integrity and in primates for DNA integrity. In rodents, there is a trend in the viability of these cells to be superior in slow freezing, although the small number of animals used requires discretion. Other authors have obtained opposite findings, being better vitrification for spermatozoa or even without differences between both methods (14, 21). Such discrepancies could be attributed to variations between cryoprotectants, media, temperature, concentration of cryoprotectants or species studied. As mentioned before, the higher dose of cryoprotectants during vitrification could be the reason, in our study, of the lower viability and DNA integrity of vitrified/warmed elongated spermatids/spermatozoa compared to those slow frozen/thawed. Other factors to consider are the cooling rate and the high temperature used for warming, since spermatozoa seem to be more sensitive to them than other germ cells (30, 33). In fact, ejaculated spermatozoa from wild ungulates have shown a poor tolerance to the fast cooling rate of vitrification (30, 31) and warming vitrified-needle samples at 50°C resulted in DNA damage in testicular spermatozoa from adult cats (33). Despite slow freezing provided better results for elongated spermatids/spermatozoa than vitrification, the viability of these testicular cells was remarkably lower than fresh ones. Perhaps, the inclusion of an equilibration step in the slow freezing protocol in a future could improve the post-thaw quality of elongated germ cells, since the equilibration period contributes to membrane lipid adaptation to cooler temperatures (34).

In rounded cells, slow freezing and vitrification yielded similar data in terms of viability, although DNA integrity decreased after slow freezing compared to vitrification. With these results in mind, vitrification seems the best method for preserving rounded germ cells, which is contrary to the results obtained in elongated germ cells. However, these findings were obtained when a general comparison was made without differentiating species according to their taxonomic group. When taxonomic groups were considered, different findings were found between them, which stands out the specific particularities of each species group for testicular cryopreservation.

The artiodactyls are the most extensive and varied group in our study. This group includes wild bovids, suids, one cervid, and one member from the tapiridae family. Since DNA integrity is a key factor for successful IVF or ICSI outcomes, although both techniques provided acceptable results in rounded cells viability and DNA integrity, the protocol used for slow freezing seems to be suboptimal for preserving the quality of testicular rounded cells in wild artiodactyls, given the lower protection of DNA. Previous studies in wild boar and collared peccary testes demonstrated comparable results between both techniques (10, 17). In fact, slow frozen/thawed and vitrified/warmed testicular tissue fragments from pigs recovered after xenotransplantation showed normal resumption of spermatogenesis (13). Conversely, slow freezing produced less damage to testicular intra-tubular cells of rusa deer, fea's muntjac, and sumatran serow than fast freezing (21). In addition, unlike vitrification, slow freezing of ram testes supported a complete differentiation of spermatogenic cells after xenografting (20). The differences between our study and others could be attributed to the huge diversity of the artiodactyl group, which might hide some species-specific differences, as earlier reported among cervid species (7, 35). Therefore, this could mean that within the same order, different species seem to manifest the need to apply different methodologies or protocols for slow freezing and vitrification to achieve better results. Something similar could be happening in the other taxonomic groups of this study. Nevertheless, our intention was to apply the same protocol of slow freezing and vitrification to all the species that constitute each taxonomic group to demonstrate the cross-species adaptability.

In non-human primates, to the best of our knowledge, there are no comparatives studies between testicular cryopreservation methods. Promising results have been previously obtained using slow freezing in diverse non-human primates, generating also healthy offspring from graft-derived spermatozoa (36, 37). Our findings agree with these earlier reports based on the greater viability and DNA integrity found in rounded cells after slow freezing in comparison to vitrification.

In carnivores, the integrity of testicular rounded cells was better maintained with the vitrification protocol than with the uncontrolled slow freezing protocol, which is in line with previous studies conducted in testicular tissues from dogs (2, 17). Notwithstanding, the results that are reported here, as in the other groups, do not necessarily mean that this taxonomic group is more sensitive to slow freezing than vitrification, it just means that the preservation method may have been suboptimal since species-specific differences were not considered due to the small number of individuals per species. A recent study has shown that testicular tissue histo-morphology and viability from black-footed ferrets, an endangered wild species, was successfully preserved by vitrification, remaining functional after culture (38). On the contrary, testicular morphology was compromised after vitrification in gray wolf and three wild felids, but not after slow freezing (9, 21). Unlike the present study, in these latter works the viability and DNA integrity of different germ cells was not evaluated.

Earlier studies on wild and domestic rodents reported that vitrification of testicular tissues works better than slow freezing (11, 15), allowing the generation of healthy progeny after culturing vitrified/warmed testicular tissues from mice (16). In our work, no differences were found between both methods in wild rodents, possibly due to the small sample size, but there is a trend in the viability of rounded cells to be higher after vitrification than after slow freezing, which highlights the similarity of the wild rodent species with the domestic ones.

The present study has some limitations. Firstly, since testicular tissues were not histologically analyzed, we were unable to describe the effect of needle immersed vitrification or uncontrolled slow freezing on testicular tissue structure. Future studies should include the histological analysis at least when rare or uncommon species are investigated to identify the morphology of different cell types. Secondly, comparison within and between species was very complicated in this preliminary work, since in some cases there was only one individual per species and for this reason the species were grouped according to their order. Different results could have been obtained if the number of individuals in each species had been larger. This would allow to study the species separately, considering their species-specific characteristics, but working with wild species makes this task difficult, since sample collection is often opportunistic and researchers have no control on the species, number of samples, age of animals, cause of death, time from death to laboratory, etc. All these factors taken together might affect the comparisons made in the present study and should be taken into account in future studies.

In summary, uncontrolled slow freezing and needle immersed vitrification are simple and easy methods that have shown encouraging results for the cryopreservation of testicular tissues from a large variety of wild species. Our preliminary findings demonstrated that the response to the testicular cryopreservation method differs between the species and cell type. Therefore, current testicular cryopreservation protocols must be adapted to our cell type interest, owing to the different cryosensity of germ cells, and also to the species of interest, since the taxonomic group can mask some species-specific particularities. Adjusting the cryopreservation method to rounded germ cells may offer major benefits, because spermatogonia and spermatocytes can differentiate into an unlimited number of spermatozoa if properly preserved and then cultured or grafted, which represents a powerful tool for the future assisted reproductive technologies.

## Data availability statement

The original contributions presented in the study are included in the article/supplementary material, further inquiries can be directed to the corresponding author.

## Ethics statement

No ethical concern was raised in relation to animal experimentation except for euthanized animals, whose procedure was evaluated and approved by the Internal Animal Welfare Committee of Zoo-Aquarium Madrid based on the EAZA (European Association of Zoos and Aquariums) Code of Ethics.

## Author contributions

JS-M, AT-D, EM-N, and CC performed study design, data analysis, and reviewed manuscript. RV, BP, BM-M, ME, and JB-B contributed to investigation and methodology. PP-F contributed to investigation and prepared the manuscript. All authors contributed to the article and approved the submitted version.
